# Numerical Simulation of Coupled Pyrolysis and Combustion Reactions with Directly Measured Fire Properties

**DOI:** 10.3390/polym12092075

**Published:** 2020-09-12

**Authors:** Khalid Moinuddin, Qazi Samia Razzaque, Ananya Thomas

**Affiliations:** Institute for Sustainable Industries and Liveable Cities, Victoria University, P.O. Box 14428, Melbourne, VIC 8001, Australia; samia.razzaque@uqconnect.edu.au (Q.S.R.); ananya.thomas@live.vu.edu.au (A.T.)

**Keywords:** CFD-based fire model, fire properties, heat release rate, mass loss, pyrolysis, combustion

## Abstract

In this study, numerical simulations of coupled solid-phase reactions (pyrolysis) and gas-phase reaction (combustion) were conducted. During a fire, both charring and non-charring materials undergo a pyrolysis as well as a combustion reaction. A three-dimensional computational fluid dynamics (CFD)-based fire model (Fire Dynamics Simulator, FDS version 6.2) was used for simulating the PMMA (non-charring), pine (charring), wool (charring) and cotton (charring) flaming fire experiments conducted with a cone calorimeter at 50 and 30 kW/m^2^ irradiance. The inputs of chemical kinetics and the heat of reaction were obtained from sample mass change and enthalpy data in TGA and differential scanning calorimetry (DSC) tests and the flammability parameters were obtained from cone calorimeter experiments. An iso-conversional analytical model was used to obtain the kinetic triplet of the above materials. The thermal properties related to heat transfer were also mostly obtained in house. All these directly measured fire properties were inputted to FDS in order to model the coupled pyrolysis–combustion reactions to obtain the heat release rate (HRR) or mass loss. The comparison of the results from the simulations of non-prescribed fires show that experimental HRR or mass loss curve can be reasonably predicted if input parameters are directly measured and appropriately used. Some guidance to the optimization and inverse analysis technique to generate fire properties is provided.

## 1. Introduction 

Under the building codes in various jurisdictions around the world (such as National Construction Code, NCC [1]), fire performance requirements in a building can be achieved either prescriptively or with a fire engineered performance solution. Prescriptively, the requirements in a Deemed-to-Satisfy design can be compared to a recipe for building design which must be followed in order to deem the building safe and compliant. This form of compliance also requires materials and forms of construction to be experimentally tested or numerically proven to withstand the standard fire curves such as ISO 834 [2]. A fire-engineered performance solution, on the other hand, analyses real fire scenarios likely to occur during the design life of the specific building. Performance-based designs are frequently used by fire engineers and researchers to take the advantage of the flexibility offered to adopt their new design concept without compromising the safety aspects required by regulations. As experimental fire tests are significantly expensive, numerical simulation is an alternative method to assess fire safety through modelling. In order to model real fire scenarios adequately, it is imperative to obtain appropriate fire properties to be used as input parameters to the model. Fire properties include parameter values related to pyrolysis reactions obtained using thermogravimetric analysis (TGA) and differential scanning calorimetry (DSC), thermo-physical properties were obtained using hot disk analyser (HDA) and flammability/combustion parameters were obtained using cone calorimetry.

The ability to reliably model any fire scenario gives fire safety engineers the confidence and power to assess the fire performance of a building accurately. One method of modelling is using computational fluid dynamics (CFD)-based fire models. Fire Dynamic Simulator (FDS) software developed by NIST which solves the Navier–Stokes equations for low speed, thermally-driven flow along with heat transfer, pyrolysis, combustion and species equations to calculate fire growth and smoke and flame propagation [3] is one such model. Modern technology provides CFD-based models with greater computing power so they can model each phase of the fire from the ignition, growth, to the flashover and decay. However, most of the validation with CFD-based fire models are either limited to solid-phase pyrolysis reaction (e.g., cone calorimeter tests with no flaming ignition) or gas phase reactions where the fire size (i.e., volatile production rate) is prescribed. It is important that validation be conducted where coupled solid and gas-phase reactions are involved. 

In previous studies [4,5,6,7,8,9,10], pyrolysis reactions in cone calorimeter tests, with no flaming ignition, have been simulated using a one-dimensional heat transfer and pyrolysis model. Simulations of pyrolysis reactions with flaming ignition using a similar one-dimensional heat transfer model were conducted in [11,12], where a fixed gas phase heat source was added to account for radiation from the flame. In [13], when fire sizes were prescribed in a CFD-based fire model, FDS prevents the gasification phase, and the gas temperatures and radiation fluxes, at various locations within a compartment, were well predicted. 

Three-dimensional CFD-based simulations of cone calorimeter tests with coupled solid and gas phase reactions have only recently been conducted [14,15,16] with directly measured fire properties. The lack of similar study may be due to the enormous computational requirements of modelling reactions at a millimetre scale. The validation of cone calorimeter experiments is vital before scaling up to a full-scale enclosure (with combustibles inside) modelling for assessing fire safety.

Marquis et al. [15] have used version 5 of FDS, whereas in [14] a user-defined function without involving the fluid momentum equation was implemented in ANSYS, a commercial finite element modelling software. As version 6 of FDS (FDS6) incorporates improved combustion, as well as large eddy simulation (LES)-based turbulence and pyrolysis sub-models, it is important that validation be conducted with FDS6 involving ‘pure’ materials. It is to be noted that FDS is the most commonly used CFD-based fire model in research and practical engineering applications. Nguyen et al. [16] have modelled using FDS6 by obtaining pyrolysis parameters using a single heating rate whereas International Confederation for Thermal Analysis and Calorimetry (ICTAC) recommendation [17] suggests that the kinetics be obtained using TGA at five heating rates, at least. Moreover, all three studies of [14,15,16] have been carried out with complex materials rather than ‘pure’ charring and non-charring materials. Drean et al. [18] modelled an intermediate scale aluminium composite material (ACM) façade test with FDS6 using a 20 mm grid and fire properties from various laboratories. There have been cone calorimeter modellings with fire properties obtained using optimization techniques [19,20,21,22]. It is to be noted that optimized properties are model specific, not generic to all models. This current study is aimed at modelling a cone calorimeter experiment with directly measured fire properties (mostly inhouse). 

Abu Bakar et al. [23,24] conducted extensive experimentation with one synthetic polymer (PMMA) and three natural polymers (pine, cotton and wool) materials with TGA and DSC at various heating rates, with a cone calorimeter at different irradiance and HDA at various temperatures. Plant-based natural polymers pine and cotton contain polymers such as cellulose, various hemicelluloses and/or lignin. Animal-based natural wool is a linear keratin polymer in which amino acids are joined together to form long polymer chains. The aim was to characterise pyrolysis, combustion, as well as the thermal and physical properties of these polymers, which are frequently found in buildings. However, the pyrolysis kinetics were not determined using the ICTAC recommendation [17]. Therefore, in this study, the TGA data of [23,24] were re-analysed in accordance with ICTAC as ICTAC recommendations were overlooked there. TGA simulations (pyrolysis only) were then conducted, followed by cone calorimeter simulations at two irradiances for these four materials with FDS6. This study ultimately aims to provide researchers and engineers with data on how well FDS6 predicts the heat release rate (HRR) and/or mass loss when directly measured fire properties were used as input. 

## 2. Fire Properties

Pyrolysis and combustions occur simultaneously in a fire thereby making it a complex process. Pyrolysis is the process of a solid transforming into the gaseous phase when the molecules are broken down into different sized molecules. It is an endothermic process controlled by many chemical reactions which are a function of temperature [25]. Pyrolysis parameters include chemical kinetics, heat of reaction (HoR) and the char yield of the material. The chemical kinetics of the material are determined by three parameters known as the kinetic triplet: (1) activation energy, *E* (kJ/mol) representing the minimum amount of energy required to start a chemical reaction, (2) a pre-exponential factor or the frequency factor, *A* (1/s for first-order reaction) accounting for the orientation and the frequency of the collisions between molecules and (3) the reaction order, *n*. The most commonly used equation to express the kinetic reactions of a material is the Arrhenius equation (Equation (1)). The Arrhenius equation is also in terms of the reaction constant, *k* (1/s for first-order reaction) and *R*, the universal gas constant:

(1) $$k=Ae^{-\frac{E}{RT}}$$

Combustion parameters consist of the effective heat of combustion, smoke yield, char residue, as well as the soot, CO and CO_2_ yield [24]. The thermo-physical properties required to model coupled pyrolysis and combustion reaction are density, thermal conductivity, specific heat capacity, absorption coefficient and emissivity. The pyrolysis, combustion and thermo-physical parameters are together often called fire properties. Kinetic triplets or parameters are obtained by postprocessing raw data from the TGA data and the process is described below.

## 3. Kinetic Parameters of PMMA, Pine, Cotton and Wool

The TGA is an analytical technique used to experimentally investigate the thermal decomposition of materials and understand the pyrolysis of materials. The TGA instrument monitors the mass loss of a material of known quantity as a function of time and/or temperature. This thermal decomposition data are then made to undergo some post processing for obtaining the kinetic parameters of the reaction. Over the years, many methods have been identified for extracting kinetic data from the TGA, however, they can be broadly divided into two main types, namely, a model-fitting or optimization method and model-free techniques. In the former method, an appropriate model which provides the best statistical fit was used to determine the kinetic parameters of the reaction. The latter technique, which is also known as the isoconversional method, requires several kinetic curves for carrying out the analysis. 

TGA analysis can be performed at a series of constant temperatures, isothermally and also non-isothermally, under different heating rates [26]. The output of the TGA analysis provides the sample mass recorded as a function of temperature for a specific heating. The mass loss with respect to temperature, i.e., a fraction of conversion *(*α*)* versus the temperature data, is used for determining chemical kinetic factors such the pre-exponential factor, *A*, activation energy, *E* (kJ/mol) and the reaction order, *n.* These can be used as inputs for pyrolysis modelling. In this method, α is determined as

(2) $$\alpha =\frac{m_{t}\;-\;mf}{m_{i}\;-\;\;mf}$$

where $m_{i}$ is the initial mass and $m_{f}$ is the final mass and $m_{t}$ is the mass measured at a given temperature. It is often assumed that the pyrolysis reactions take place as per the Arrhenius equation (Equation (3)) to express the kinetic reactions of a material:

(3) $$\frac{d\alpha }{dT}=\frac{A}{\beta }e^{\left(-\;\frac{E}{RT}\right)}{\left(1\;\;-\;\;\alpha \right)}^{n}$$

where (*d*α*/dT)* is the pyrolysis rate, and *β* is the heating rate (K/s) in the TGA experiment, *T* is the sample temperature (*K*) and *R* is the universal gas constant (8.31 × 10^−3^ kJ/mol.*K*). Equation (3) is used in FDS by default. By applying appropriate data reduction methodology on the TGA data *A*, *E* and *n* can be obtained.

As per the ICTAC recommendation [17], it is important to determine the activation energy (*E*) over multiple (at least five) heating rates rather than one heating rate. This ensures that the *E* value is not heating rate specific. Equations for the non-isothermal kinetic analysis of TGA data is expressed by kinetic expressions for isothermal experiments. For data where α, *T* (temperature), f(α) and β are known, Equation (4) can be used to represent one of the differential methods for TGA, where f(α) is the reaction model and $\beta =\frac{dT}{dt}$ heating rate [27]:

(4) $$\frac{d\alpha }{dt}=\left(\frac{A}{\beta }\right)e^{-\;\frac{E_{a}}{RT}}\times \;f\left(\alpha \right)$$

**Figure 1:**
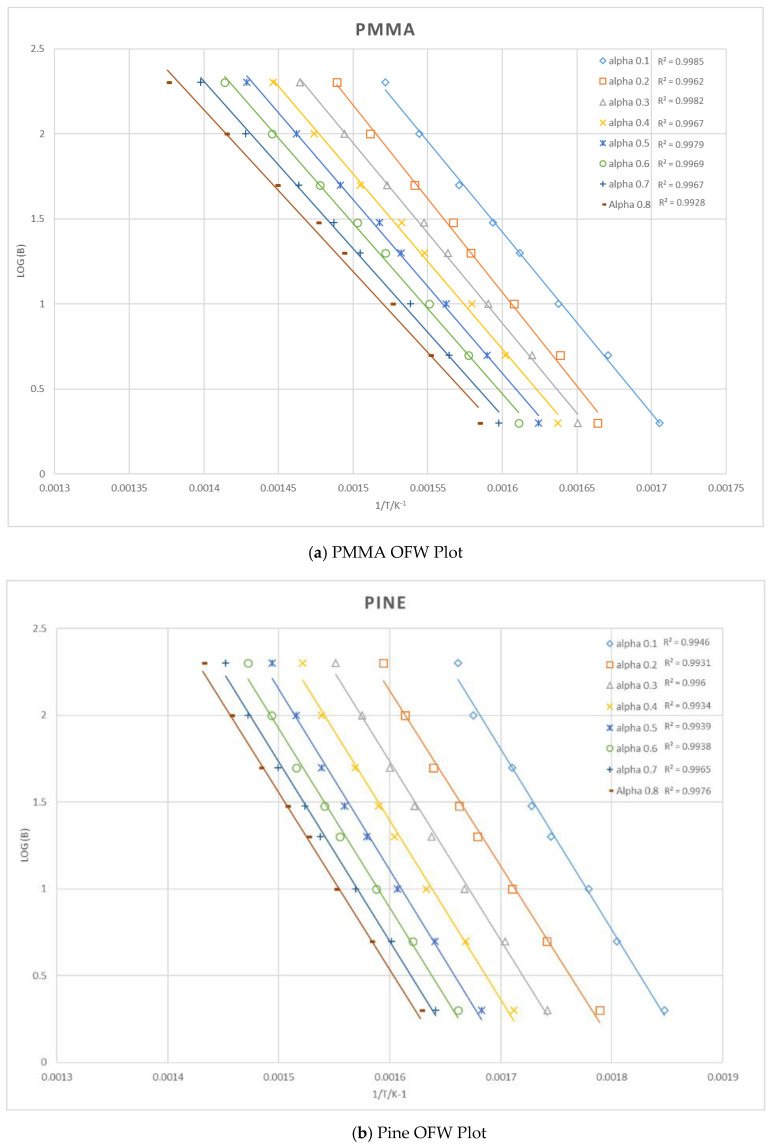

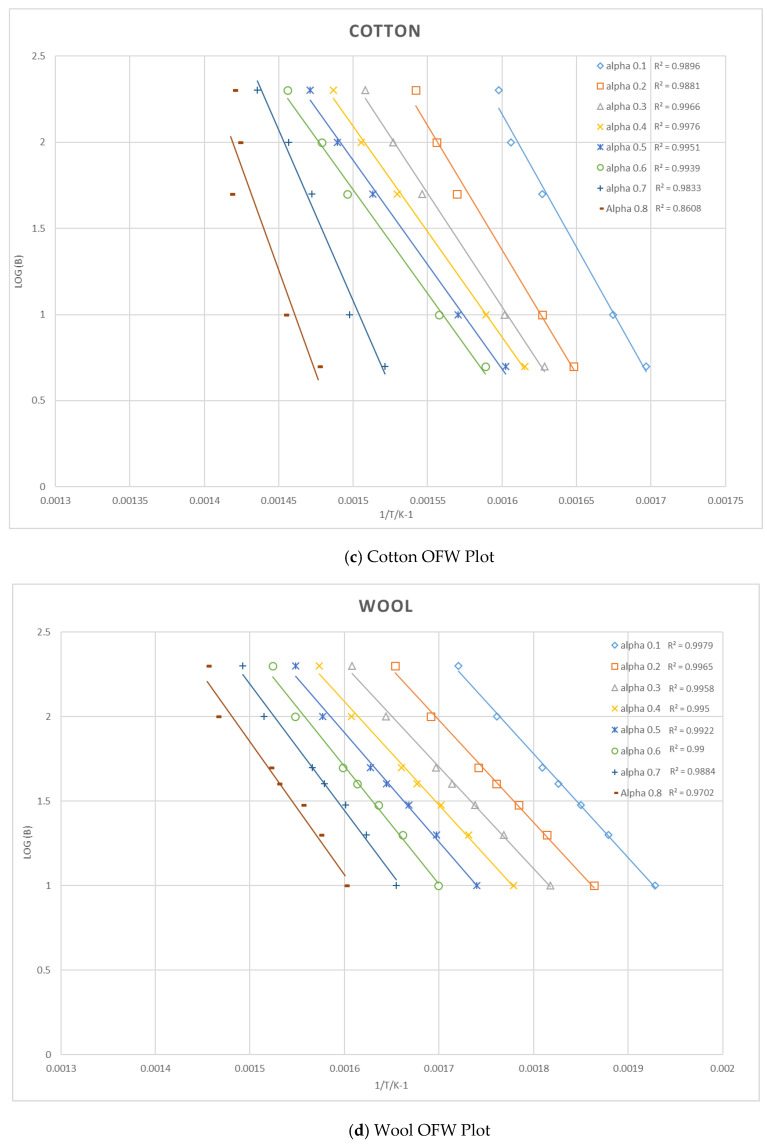
The Ozawa–Flynn–Wall (OFW) method plots for the TGA tests with PMMA, pine, wool and cotton.

The activation energy, *E*, can be found as the gradient, when the $Log_{10}$ *β* versus 1/*T* is plotted as stated by Ozawa [28] and Flynn and Wall [29]. This model is known as the Ozawa–Flynn–Wall (OFW) method and is an iso-conversion procedure called ‘integral iso-conversion method [30]. The independently developed iso-conversional calculation takes the natural logarithm of the non-isothermal rate law and uses Doyle’s approximation for temperature integral [28]. It is based on multiple plots of log_10_ heating rate (*β*) against 1/$T_{\alpha }$ for each heating rate at a fixed degree of conversion, α, which should give a straight line [28]. The gradient of this is equal to $–0.4567\left(\frac{E_{a}}{R}\right)$ which is re-arranged to find the activation energy [28]. This is based on the equation below as given by the OFW method [29]:

(5) $${Log}_{10}\beta \;=\;-\;2.315+{Log}_{10}\;\left({AE}_{a}/R\right)-{\;Log}_{10}\;g(\alpha )\;–0.4567\left(\frac{E_{a}}{{RT}_{\alpha }}\right)$$

where α is calculated at each temperature and defined as the weight fraction or factor of conversion of polymer which reacted as given in Equation (2). Prior to determining the weight fraction from the TGA (Mettler-Toledo Corporation, Greifensee, Switzerland) instrument data, the moisture and char of each material were removed. The mass fraction values, α, ranging from 0.1 to 0.8, were used in the OFW analysis and the α at the maximum value of $\frac{d\alpha }{dt}$ across each heating rate was averaged and used to determine the activation energy, *E* [27]. Figure 1 shows the OFW method plots used to find the activation energy of each material. The gradient of each line is then equated to −0.4567(*E_a_*/*R*) to solve for *E*.

**Figure 2:**
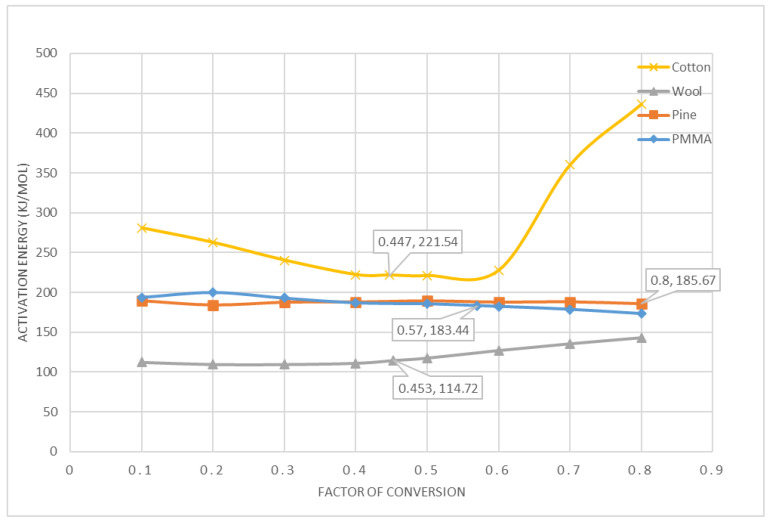
Variation of the activation energy for the materials using the OFW method.

Figure 2 shows the activation energy for each factor of conversation for the materials analysed using the OFW method. It can be seen that pine and PMMA have relatively constant activation energy over the pyrolysis reaction while for cotton varies the most followed by for wool. The *α* and *E* values in kJ/mol related to the maximum *dα/dT* are shown as *α, E* on each profile.

The pre-exponential factor, *A*, was then found based on the *E* of each material with the aid of TGAnalysisV7.0 software (developed by Bigger et al. [26]). This software reconstructs TG graphs from pre-determined kinetic models of Arrhenius parameters, using an iterative arithmetic technique. It uses a model identification algorithm and provides a pre-exponential factor for various kinetic models of acceleratory, sigmoidal and decelerator kinetic mechanisms. Table 1 lists the governing equations of the various kinetic models employed by TGAnalysisV7.0 software. F1 First order is the model used in FDS. The main advantage of using the software is that it serves as a direct method of solving the Arrhenius integral without using complex mathematical approximations or calculations for processing the TGA data. It is to be noted that fabrics data in [24] shows multiple peaks of *dα/dT,* especially for cotton. Instead of multiple sets of kinetics, a single “effective” set of kinetics is determined in this study.

Each of these pre-exponential factors has then been included into a classical model, (by solving Equation (3) with a forward differencing method; also discussed in Section 5.1), to determine which kinetic model (listed as Equation in Table 1) provides the closest fit to the experimental data. *A* for each kinetic model was found and then used into the classical model. The final *A* value is taken, when the classical model matched closest to the experimental (TGA) data. For pine “R1 Contracting area”, for wool “F1 First order” and for cotton the “A2 Avrami–Erofeev Model” kinetic model best fit the data. On the other hand, PMMA configured best with “E1 Exponential law”.

Table 2 summarises the kinetic parameters for each material analysed. The HoR values were directly taken from [23,24,31]. The reaction order, *n*, for the purpose of this study, was considered a unity (1).

## 4. Flammability and Thermo-Physical Parameters

The flammability properties, as well as the moisture and char residue of each material, can play an important part in the time to ignition and HRR output in each material simulation. Cone calorimeter (Fire testing technology, East Grinstead, UK) experiments were conducted by Abu Bakar et al. [23,24]. The CO yield, soot yield, and char residue parameter from these studies for two irradiance levels 50 and 30 kW/m^2^ are listed in Table 3. The effective heat of combustion (EHoC) for non-charring material (PMMA) was also taken from [23]. For charring materials (pine, cotton and wool), a different approach was undertaken for EHoC. The cone calorimeter experiments also give the total mass loss and total heat release data. The EHoC for charring materials, listed in Table 3, were obtained by dividing the total heat release (kJ) with total mass loss (kg). However, given the small mass sample of cotton and wool, the HRR and total heat release measured may not be accurate and there may be up to 40% uncertainty. Additionally, the presence of moisture can affect the effective heat of combustion which in turn affects the simulation outcome. Therefore, during the numerical modelling (with FDS), the measured EHoC was increased gradually until ignition occurred. Moisture fractions were taken from the TGA data of [23,24].

Cone calorimeter experiments also measure the time series of HRRPUA (HRR per unit area) and mass loss. This time series data were taken as benchmarks for the validation of the coupled pyrolysis–combustion model, FDS in this study. Data presented in Table 2 and Table 3 were used as input into the FDS for the cone calorimeter simulation.

The thermo-physical properties related to heat transfer used as input to FDS modelling for cone calorimeter simulation are listed in Table 4. Thermal conductivity data were taken from Abu Bakar et al. [23,32] which were measured using a hot disk analyser (HDA) and specific heat data were taken from [33] which was measured using a DSC. Thereby, most data used for numerical simulation (Table 2, Table 3 and Table 4) were obtained in house (Institute for Sustainable Industries and Liveable Cities, Victoria University). The emissivity and absorption coefficient of PMMA are the only literature data used as input in the simulations. The emissivity for charring materials was taken as a unity as the surface blackens soon after the ignition. It is one of the rare studies where almost all the material properties were collected in-house.

## 5. Numerical Analysis of Pyrolysis

### 5.1. Classical Theory (Arrhenius Equation)

**Figure 3:**
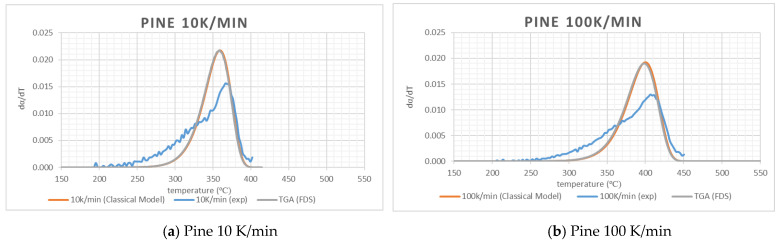

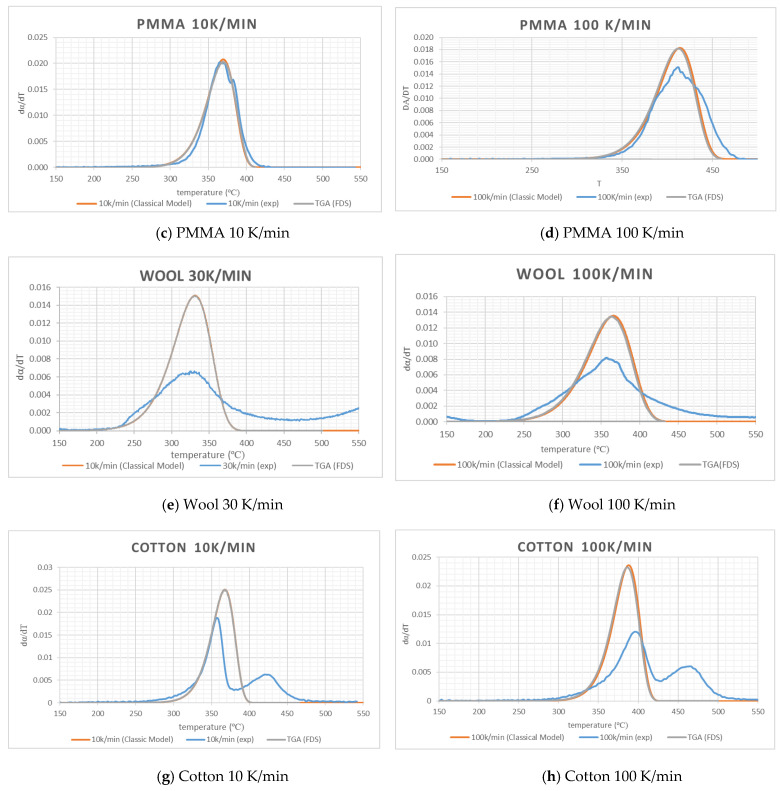
Experimental data and simulation data using kinetics data.

To check the validity of the values of *A, E* and *n* of Table 2, *dα/dT* was calculated by solving Equation (3) with a forward differencing method (temperature step was taken as 0.25 °C) incorporating kinetic values from Table 2. The calculated profile will be termed as classical here. In Figure 3, it can be observed that the profiles obtained using the classical method agrees reasonably well with the experimental results for PMMA and pine. This gives further confidence in the quality of the experimental data as well as the adequacy of the OFW method. Due to use of a single “effective” set of kinetics for cotton and wool, the differences are significant. However, the area under the curve for cotton and wool are also similar to the experimental data.

### 5.2. TGA Modelling Using FDS

To check how well the kinetics values serve as input for the numerical simulation of pyrolysis, FDS version 6.2 as used in [37] was used to perform the computation. In this version, the combustion model was less computationally demanding to obtain grid convergence. In TGA simulations, FDS was asked to simulate the solid phase only using kinetics from Table 2 and a “lumped mass” sample was heated by radiation only. Nominal values of thermal conductivity, specific heat and density are specified. FDS default values of emissivity, absorption coefficient and HoR were used. DTG (dα/dT) versus the temperature profiles were calculated using sample masses and temperatures. The results from the FDS simulation of TGA tests at 10 K/min (30 K/min for wool) and 100 K/min heating rates are presented in Figure 3. It can be observed that the FDS results match the classical model and it gives confidence to the FDS model. The difference with the TGA experiments is the same as the difference between the experimental and classical model results. It is to be noted that the main focus in TGA modelling is on the phenomenon of the pyrolysis of materials. A detailed study of char carbonization is out of the scope of this study.

## 6. Numerical Simulation of Cone Calorimeter

### 6.1. Model Set-Up

To simulate the cone calorimeter experiments, a domain of 0.2 m × 0.2 m area and 0.7 m height was created. This domain was selected after some domain sensitivity analysis to ensure that all flames were captured. All sides of the domain were open. The sample was modelled as an obstruction of 0.1 m × 0.1 mm placed horizontally and centrally near the bottom of the domain. The thicknesses were 0.025, 0.0188, 0.00063 and 0.001 m for PMMA, pine, cotton and wool, respectively. The top face of the obstruction represented the fuel surface and the other faces were modelled as steel sheet. In all cases, the back of the sample was considered to have a thin steel plate (0.0007 m for PMMA and pine; 0.0006 m for fabrics) to represent the heat transfer to steel cases or steel meshes and this plate was insulated. Note that in FDS, conductive heat transfer is one-dimensional, and this arrangement may be the best representation as the samples were encased or near insulated during the experiments with only the sample face exposed. The burning of a sample is simulated with an external heat flux to represent the effect of the cone heater without including the cone itself.

For conductive heat transfer, fine grid resolutions were used for solid objects by setting CELL_SIZE_FACTOR = 0.5 and perfectly uniform meshes were used. This resulted in 153 layers for PMMA, 101 for pine, 4 for cotton and 6 for wool. The layer thicknesses were in the range of 0.00016–0.00019 m. The solid-phase solution was updated at every time step (same as the gas phase).

**Figure 4:**
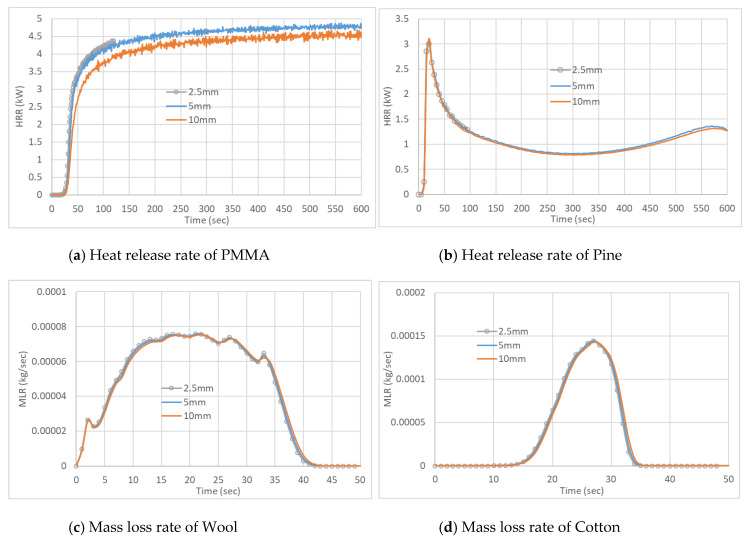
Gas-phase grid convergence analysis.

Cuboid grid sizes were selected for the gas phase reaction. Grid sensitivity results are presented in Figure 4. It can be observed that each cell size measured 0.005 m × 0.005 m × 0.005 m, which is sufficient for all materials. It appears that a 0.01 m × 0.01 m × 0.01 m cell size may be sufficient for charring materials.

**Figure 5:**
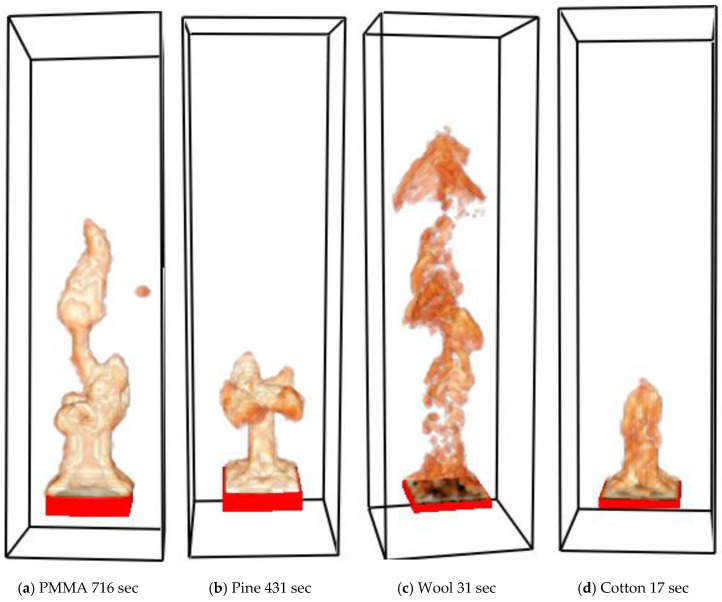
Modelled flaming combustion of four materials within domains. Visualization is done by using FDS’s companion software Smokeview based on volumetric heat release rate (HRR) in the gas phase.

Representative flaming combustions for all four materials within the simulation domain are shown in Figure 5.

### 6.2. Results

**Figure 6:**
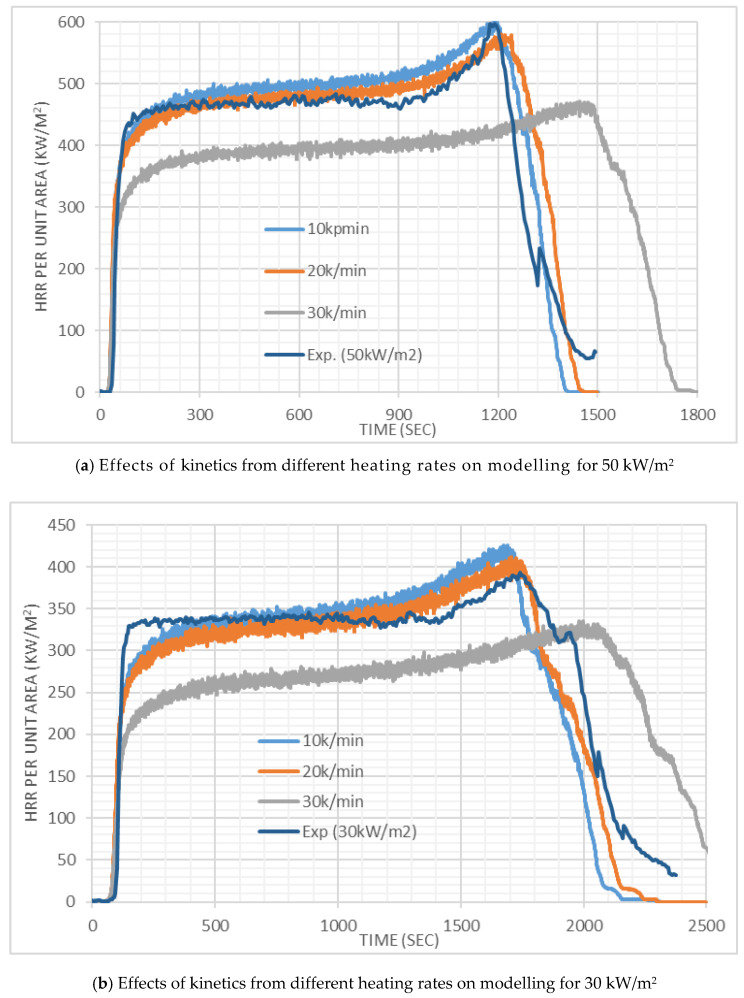
Heat release rate results for the cone calorimeter tests with PMMA.

The HRR results from the FDS simulations for PMMA at 50 and 30 kW/m^2^ irradiation are compared with the experimental results in Figure 6. It can be seen that at 50 kW/m^2^ irradiation, with *A* and HoR values corresponding to the heating rates of 10 and 20 K/min, the simulated results match the experimental outcome quite well. Better results are obtained with values corresponding to 20 K/min. At 30 kW/m^2^ irradiation, with 10 and 20 K/min values, initially the HRR was under predicted up to ~500 s, then well predicted up to ~1250 s and then over predicted. Overall, a good prediction was obtained with 20 K/min values at this irradiation. At both irradiations, times of ignition match well with the experiments of three sets of values presented here. Figure 6 also shows that for the PMMA simulations, the results with *A* and HoR values obtained at lower heating rates yield higher HRR values. Higher HoR values at a higher heating rate (explained in detail in Abu Bakar et al. [23]) is likely to be the primary reason. It can be seen from the cotton simulations (Figure 9), where HoR and *E* values are fixed, with the variation of *A*, no significant difference was observed.

**Figure 7:**
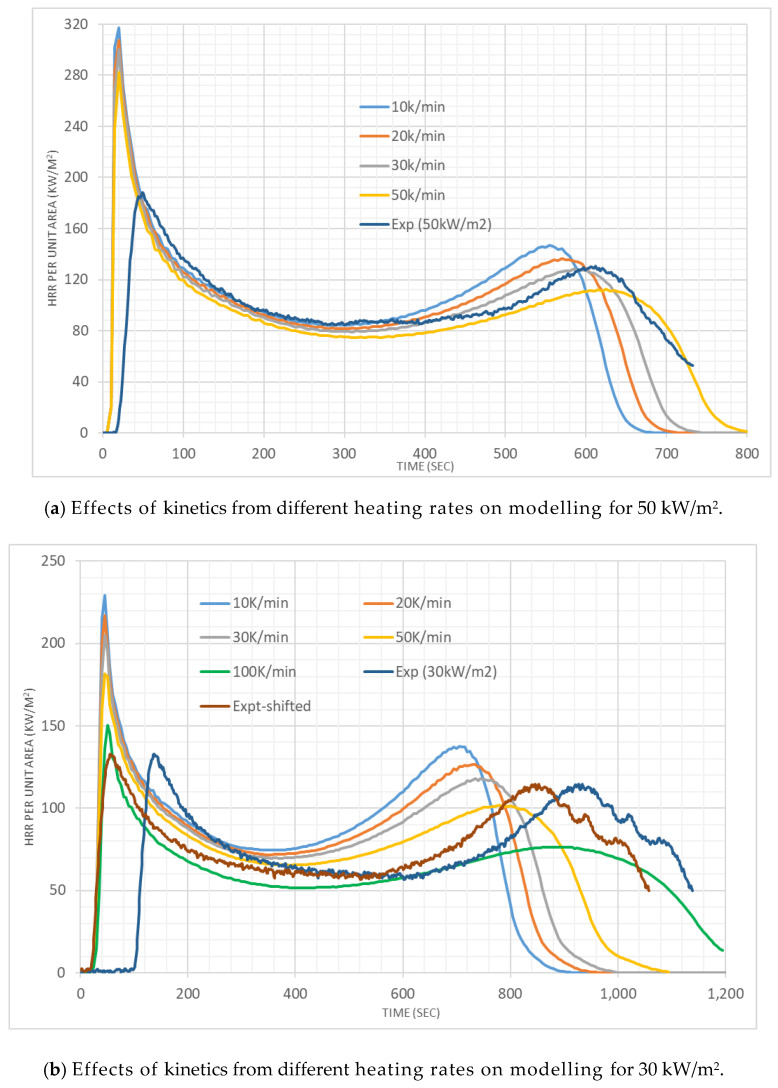
Heat release rate results for the cone calorimeter tests with pine.

In Figure 7, at 50 and 30 kW/m^2^ irradiation, the HRR results from the FDS simulations for pine are compared with experimental results. Generally, we see the same sort of trend of decreased HRR with the increased HoR associated with higher heating rates. This implies that the kinetics parameters obtained at high heating rates provide less conservative estimations of HRRPUA. The ignition occurs 10 s earlier in the simulation compared to the experiment at 50 kW/m^2^ irradiation. The first peak is also significantly higher in the simulation. The first peak appears to correspond with the initial pyrolysis from the top layer. However, after ~60 s the simulated HRR values are closer to experimental values and the second peak value is also close. The second peak is associated with the thermal wave in the fuel hitting the insulation bottom [38,39]. Overall, *A* and HoR values associated with 30 K/min give the closest result. At 30 kW/m^2^ irradiation, ignition occurs ~60 s earlier in the simulation, which is quite significant. A comparison with HRR time series is made by shifting the experimental HRR time series by 60 s. Simulations, at this irradiation, yield overall higher HRR which may be considered conservative in relation to fire severity [40]. However, the simulation with *A* and HoR values associated with 100 K/min provide a lower HRR and a longer burning duration.

**Figure 8:**
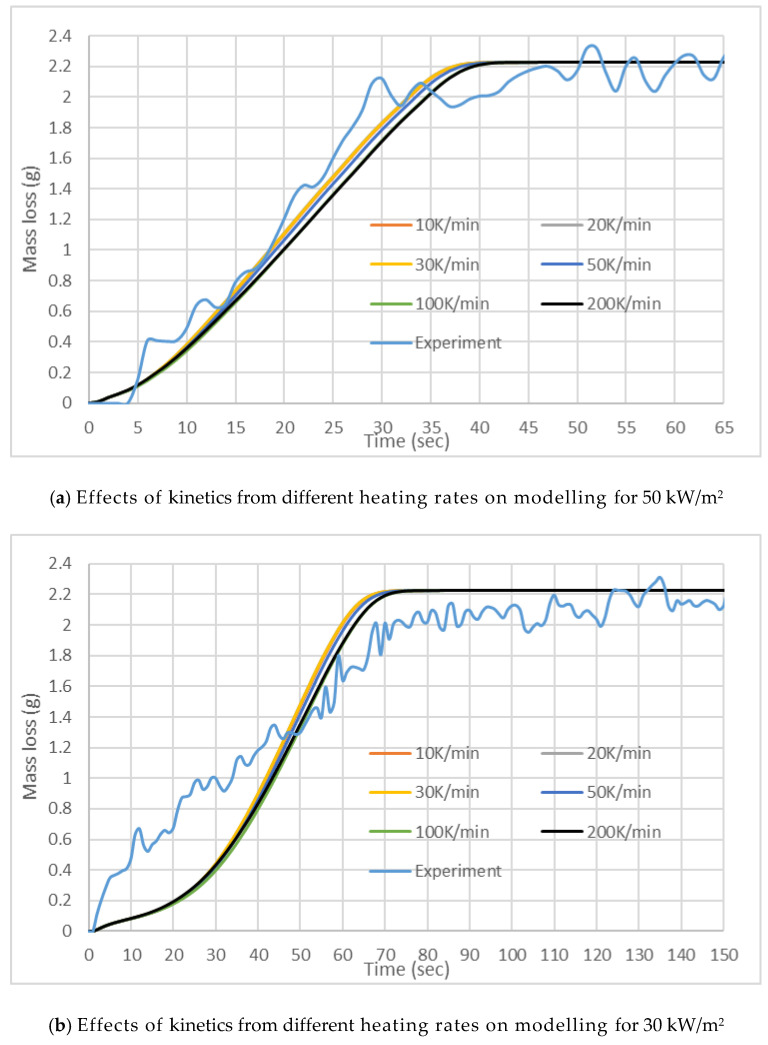
Mass loss results for the cone calorimeter tests with wool.

Given the uncertainty with the HRR measurement and EHoC determination with the small mass fabrics sample (Section 4), instead of HRR time series, mass loss time series data are compared for cotton and wool. Figure 8 shows the mass loss curves generated by FDS simulations compared to the experimental results for wool. At 50 kW/m^2^ irradiation, the simulation results are quite close to the experimental results. On the other hand, when the sample is exposed to a lower heat flux of 30 kW/m^2^, the simulation results are underpredicted during most part of the burning. The sensitivity of charring properties in wool can have a significant effect on the numerical model as wool char properties could not be measured. We used the properties of pine char. As shown in Table 3, the moisture content of 6% and char of 3.8%were modelled for Wool in line with the experimental results.

**Figure 9:**
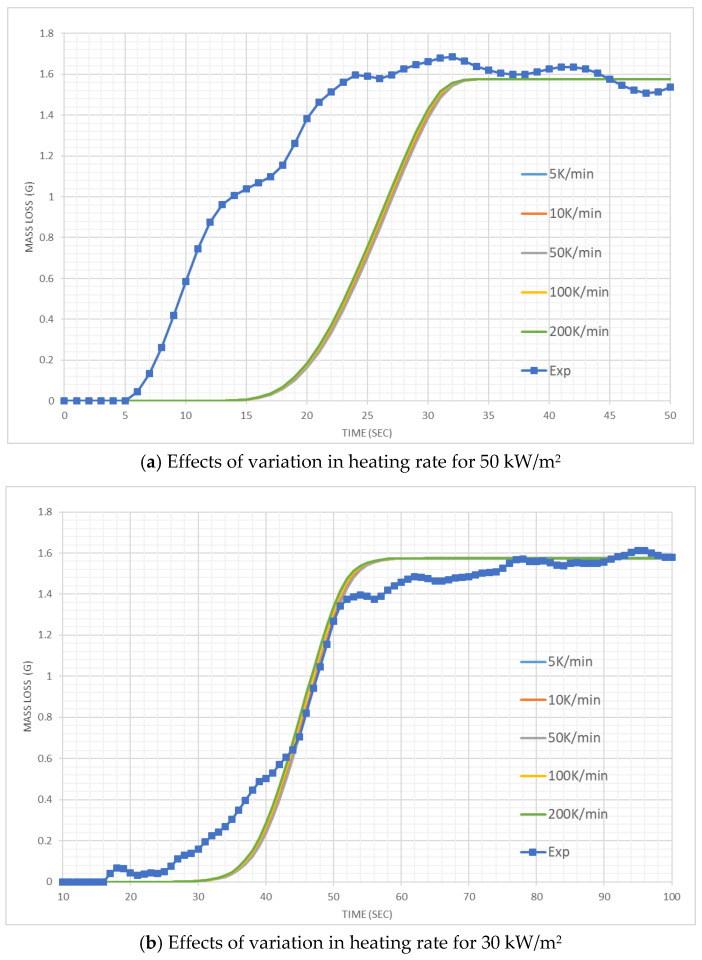
Mass loss results for the cone calorimeter tests with Cotton.

Figure 9 reveals the mass loss observed for cotton in this study. At both irradiation, simulated mass loss curves have similar shapes to the corresponding experimental curves. However, for 50 kW/m^2^ irradiation, ignition and thereby, a simulated curve occurred ~14 s later than the experimental curve. For the lower irradiation, closer simulation results were obtained. Besides charring properties, physical properties like porosity could play a role in the simulation. This property is not used in FDS [22]. Furthermore, for the pyrolysis of cotton, we considered a single “effective” reaction as opposed to two reactions.

When charring and physical properties cannot be measured, literature values for similar materials can be used or optimization methods such as a genetic algorithm [19,20,21,22] can be adopted. However, for the optimization method, directly measured fire properties can be kept unchanged and the rest of the properties can be optimised.

Furthermore, it is to be noted that TGA/DSC analyses only provide mass loss data with respect to the time/temperature of only a mg of the sample at a specific heating rate, it does not incorporate the effect of convective heat transfer and conduction that essentially occurs in a real fire scenario. Hence, the use of TGA/DSC data for large scale fire simulations should be dealt with cautiously. Optimization and inverse analysis can play a role in making necessary calibrations, especially with contemporary construction products which are quite complex. However, the directly measured values should be the base values for the optimizations.

## 7. Conclusions

In this study, directly measured fire properties were used as input for coupled solid and gas phase reaction simulations to predict the HRR and/or mass loss measured in cone calorimeter experiments. To model pyrolysis, following ICTAC recommendation [17], the OFW method [29] was used to determine the activation energy across multiple heating rates. Then, the pre-exponential factor was found for each heating rate and the reaction order was taken to be one. TGA modelling was conducted using the “classical” model by the forward time stepping of Equation (3) and also by using FDS. The results were found to be identical between two models, however, there were differences with the experimental data while the differences were the least for PMMA.

Multiple cone calorimeter simulations were carried out at the same irradiance by altering the values of the pre-exponential factor and the HoR relevant to changing heating rates. Two irradiance levels were used: 50 and 30 kW/m^2^. The results of the experimental HRR compared to the FDS modelled HRR show that the PMMA experiments can mostly be accurately represented by directly measured fire properties. The simulations of pine, wool and cotton also show close comparisons between the experimental and simulation results, however, not to the same degree as PMMA as in each case the result varied with the incident heat flux. For pine, at 30 kW/m^2^ irradiance, the ignition occurred ~60 s earlier and a higher HRR was yielded. From a fire safety modelling perspective, it may not be problematic due to the conservative fire severity predictions. For cotton, at 50 kW/m^2^ irradiance, ignition occurs later than in the experiment. The use of a thin sample, uncertainty with char properties, “effective” single pyrolysis reaction assumption, etc., can be the reasons for the difference. Furthermore, porosity is not modelled in FDS. However, the shapes of the profiles are similar between the experiment and simulation. Overall, HoR measured at 20 K/min for non-charring and at 30 K/min for charring materials provide the closest result with the experimental findings. For further improvement, it was recommended that for uncertain fire properties, the optimization method be used, keeping directly measured fire properties unchanged and the porosity model be implemented in FDS. Optimization and inverse analysis can also play a role in making calibrations of data from mg scale thermal analysis (TGA/DSC) for large-scale fire simulations, keeping the directly measured values as the base values. A further study with the cone calorimeter testing of thicker fabric samples can be carried out. Char properties of specific fabrics can be determined as well.

It is important to understand the effects of irradiance on combustion parameters. It is likely that varying levels of moisture content in charring materials also resulted in the variation of results. The presence of moisture can have an effect on the effective heat of combustion which in turn affects the simulation outcome. Variation in char development with different irradiance heat flux can also be more prominent in dense thick samples, as the moisture evaporation and char formation are not always uniform through the sample depth. PMMA being the only non-charring material investigated, supports the hypothesis that it is more difficult to accurately simulate experimental HRR for different heat fluxes using constant fire properties in charring materials.

Efforts may be undertaken to measure the fire properties varying with heating rate, incident heat flux and time. It is expected that if the values of all these parameters relevant to changing heating rates are used as the fire grows or burns out, more accurate predictions could be obtained.

It is to be noted that the experiments involved TGA and cone calorimeter and these test conditions were modelled. In this study, chemical kinetics, HoR, flammability parameters, etc. are obtained from these bench scale tests. The modelling needs to be tested against experiments with medium-scale samples. Such medium-scale experiments are the subject of future studies.

## Figures and Tables

**Table 1:** Governing Equations of the Various Kinetic Models Employed in the Software.

Sl. No.	Kinetic Model	Equation (1/*df(α)/dα)*
1	P1 Power Law	α^1/n^
2	E1 Exponential law	ln(α)
3	A2 Avrami–Erofeev Model	[−ln(1 − α)]^1/2^
4	A3 Avrami–Erofeev Model	[−ln(1 − α)]^1/3^
5	A4 Avrami–Erofeev Model	[–ln(1 − α)]^1/4^
6	B1 Prout–Tompkins	[−ln(α/(1 − α))] + C
7	R1 Contracting area	1 − (1 − α)^1/2^
8	R3 Contracting volume	1 − (1 − α)^1/3^
9	D1 One dimensional	α^2^
10	D2 Two dimensional	(1 − α)ln(1 − α) + α
11	D3 Three dimensional	[1 − (1 − α)^1/3^]^2^
12	D4 Ginstling–Brounshtein	(1 − 2α/3) − (1 − α)^2/3^
13	F1 First order	−ln(1 − α)
14	F2 Second order	1/(1 − α)
15	F3 Third order	1/(1 − α)^2^

**Table 2:** Activation Energy (E) and Pre-exponential Factor (A) at Each Heating Rate and Heat of Reaction (HoR).

Material	Heating Rate	E (kJ/mol)	A (1/s)	HoR (kJ/kg)	Material	Heating Rate	E (kJ/mol)	A (1/s)	HoR (kJ/kg)
Pine	10 K/min	185.67	2.05 × 10^13^	97.4	Cotton	5 K/min	221.54	2.06 × 10^16^	385
	20 K/min		2.09 × 10^13^	137.2		10 K/min		1.84 × 10^16^	
	30 K/min		2.04 × 10^13^	172.5		50 K/min		1.76 × 10^16^	
	50 K/min		2.13 × 10^13^	254.3		100 K/min		2.05 × 10^16^	
	100 K/min		2.13 × 10^13^	357.8		200 K/min		2.16 × 10^16^	
	200 K/min		2.55 × 10^13^	461.4	Wool	10 K/min	114.72	1.45 × 10^8^	314.8
PMMA	10 K/min	183.44	7.25 × 10^12^	1747.2		20 K/min		1.53 × 10^8^	346.3
	20 K/min		7.79 × 10^12^	2019.9		30 K/min		1.57 × 10^8^	377.7
	30 K/min		7.94 × 10^12^	2335.1		40 K/min		1.45 × 10^8^	409.2
	50 *K* /min		7.6 × 10^12^	3120.6		50 K/min		1.45 × 10^8^	440.7
	100 K/min		6.9 × 10^12^	6443.3		100 *K* min		1.36 × 10^8^	598.2
	200 K/min		6.26 × 10^12^	27468.4		200 K/min		1.84 × 10^8^	913.2

**Table 3:** Flammability Properties, Moisture and Char Residue.

Material	Irradiation	EHoC (kJ/kg)	CO Yield (kg/kg)	Soot Yield (kg/kg)	Moisture (Fraction)	Char Residue (Fraction)
Pine	30 kW/m^2^	11,210	0.007	0.006	0.035	0.105
	50 kW/m^2^	11,210	0.007	0.006	0.035	0.126
PMMA	30 kW/m^2^	21,295	0.007	0.14	-	-
	50 kW/m^2^	21,295	0.007	0.14	-	-
Cotton	30 kW/m^2^	8927	0.013	0.022	0.012	0.025
	50 kW/m^2^	5363 + 40% ^1^	0.013	0.022	0.012	0.025
Wool	30 kW/m^2^	6300 + 28% ^1^	0.01	0.039	0.06	0.038
	50 kW/m^2^	7687 + 5% ^1^	0.01	0.039	0.06	0.038

^1^ % increase for Fire Dynamic Simulator (FDS) modelling.

**Table 4:** Specified Thermo-Physical Properties.

Material	Properties	Value	Unit	Value	Material
Pine	Thermal Conductivity	0.168; 20 > T 0.0002T + 0.1649; 20 ≤ T ≤ 225 0.2; T > 225	W/m/K	0.1945	PMMA
	Specific heat	0.756; 25 > T 0.004T + 0.6544; 25 ≤ T≤240 1.614; T > 240	kJ/kg/K	1.47	
	Emissivity	1		0.85 [34]	
	Absorption Coefficient	Default	m^−1^	2700 [35]	
	Density	403	kg/m^3^	1210	
Char ^1^	Thermal Conductivity	0.069; 20 > T 0.0001T + 0.0661; 20 ≤ T ≤ 225 0.102; T > 225	W/m/K	48; 20 > T −23.107T + 1139; 20 ≤ T ≤ 677 30; T > 677	Steel [36]
	Specific Heat	0.927; 25 > T 0.0028T + 0.8587; 25 ≤ T ≤300 1.697; T > 300	kJ/kg/K	0.45; 20 > T 6 × 10^−07^ T^2^ + 0.0002T + 4463; 20 ≤ T ≤ 200 0.85; T > 677	
	Emissivity	1		0.9	
	Density	110	kg/m^3^	7850	
Wool	Thermal Conductivity	0.0846; 20 > T 1× 10^−06^ T^2^ − 0.0002T + 0.0882; 20 ≤ T ≤ 200 0.0882; T > 200	W/m/K	0.142; 20 > T 0.0002T + 0.1378; 20 ≤ T ≤ 200 0.178; T > 200	Cotton
	Specific Heat	1.773; 20 > T 9 × 10^−06^ T^3^ − 0.000355T^2^ + 0.04237T − 0.06137; 20 ≤ T ≤ 275 3.583; T > 275	kJ/kg/K	1.672; 20 > T 0.0024T + 1.6238; 20 ≤ T ≤ 300 2.344; T > 300	
	Emissivity	1		1	
	Absorption Coefficient	50000	m^−1^	50000	
	Density	220	kg/m^3^	254	

^1^ These are properties of pine char. As thin cotton and wool samples were burned, char could not be collected for hot disk analyser (HDA) measurements. Therefore, for the cotton and wool simulations, the properties of pine char were used.
